# Deregulation of cancer-stem-cell-associated miRNAs in tissues and sera of colorectal cancer patients

**DOI:** 10.18632/oncotarget.27411

**Published:** 2020-01-14

**Authors:** Cristiano Farace, Andrea Pisano, Carmen Griñan-Lison, Giuliana Solinas, Gema Jiménez, Marina Serra, Esmeralda Carrillo, Fabrizio Scognamillo, Federico Attene, Andrea Montella, Juan Antonio Marchal, Roberto Madeddu

**Affiliations:** ^1^ Department of Biomedical Sciences, University of Sassari, Sassari, Italy; ^2^ National Institute of Biostructures and Biosystems, Rome, Italy; ^3^ Biopathology and Regenerative Medicine Institute (IBIMER), Centre for Biomedical Research (CIBM), University of Granada, Granada, Spain; ^4^ Instituto de Investigación Biosanitaria (ibs.Granada), Granada, Spain; ^5^ Bio-Health Research Foundation of Eastern Andalusia - Alejandro Otero (FIBAO), Granada, Spain; ^6^ Department of Human Anatomy and Embryology, Faculty of Medicine, University of Granada, Granada, Spain; ^7^ O.U. of Surgery I (Surgical Pathology), A.O.U. Sassari, Sassari, Italy

**Keywords:** colorectal cancer, metastasis, cancer stem cell, microRNA, biomarker

## Abstract

Colorectal cancer (CRC) is a deadly tumour in Western countries characterized by high cellular/molecular heterogeneity. Cancer stem cells (CSC) act in cancer recurrence, drug-resistance and in metastatic epithelial-to-mesenchymal transition. microRNAs (miRNAs) contribute to cancer is increasing, and miRNA roles in CSC phenotype and fate and their utility as CRC biomarkers have also been reported. Here, we investigated miR-21, miR-221, miR-18a, miR-210, miR-31, miR-34a, miR-10b and miR-16 expression in experimental ALDH^+^ and CD44^+^/CD326^+^ colorectal CSCs obtained from the human CRC cell lines HCT-116, HT-29 and T-84. Then, we moved our analysis in cancer tissue (CT), healthy tissue (HT) and serum (S) of adult CRC patients (n=12), determining relationships with clinical parameters (age, sex, metastasis, biochemical serum markers). Specific miRNA patterns were evident *in vitro* (normal, monolayers and CSCs) and in patients’ samples stratified by TNM stage (LOW vs HIGH) or metastasis (Met vs no-Met). miR-21, miR-210, miR-34a upregulation ad miR-16 dowregulation associated with the CSCs phenotype. miR-31b robustly overexpressed in monolayers and CSCs, and in CT ad S of HIGH grade and Met patients, suggesting a role as marker of CRC progression and metastasis. miR-18a upregulated in all cancer models and associated to CSC phenotype, and to metastasis and age in patients. miR-10b downregulated in CT and S of LOW/HIGH grade and no-Met patients. Our results identify miRNAs useful as colorectal CSC biomarker and that miR-21, miR-210, miR-10b and miR-31b are promising markers of CRC. A specific role of miR-18a as metastatic CRC serum biomarker in adult patients was also highlighted.

## INTRODUCTION

Colorectal cancer (CRC) is a major age-related malignant tumour, typical of Western and developing countries. Although Western countries have the record for the number of CRC cases and mortality, the trend is changing. Mortality and incidence are on the rise in developing countries. The change could be due, first of all, to the improvement of the diagnosis and prevention in the richest countries and, perhaps, also for the westernization of the developing countries with changes in the lifestyle and in the alimentary attitude. Its prevention, management and diagnosis must be improved because the age of onset has recently decreased [1]. Although this trend is highly contentious [2–5], it is quite clear that symptoms appear earlier in younger patients, who are also at greater risk of second primary cancers [6]. On the other side, in most of adult patients CRC could asymptomatically advance to high cancer staging ad metastasis, when surgery and radio- and chemotherapy are no more effective. The CRC transformation is an asymptomatic multistep process which requires a long time up to 15 years to develop, and hyperplasia and dysplasia represent two early sequential adenomatous cell phenotypes that arise before and preparatory to intestinal epithelial or gland cell transformation with metastatic potential. Therefore, diagnostic/prognostic procedures that take advantage of this valuable temporal window, as well as during cancer initiation, progression ad metastasis are strongly recommended.

Genetic analyses have clearly defined a set of gene mutations that drives the initiation of CRC, changing normal cells to adenoma cells and adenoma cells to adenocarcinoma, and the metastatic process. Their identification have opened up the way for promising gene-targeted therapies. The centrality and essentiality of the cancer cell of origin and the impact of the cancer microenvironment on cell fate decisions, including the metastasis-promoting bidirectional epithelial-to-mesenchymal transition (EMT), have also been investigated and should be key considerations in new targeted therapies [7]. Within this milieu, cancer stem cells (CSC) could not only directly involved as cancer cells of origin, but also in cancer progression and recurrence, cell heterogeneity and resistance to chemotherapy, invasion and metastasis [7–10]. Recent studies of the roles of CRC cell of origin in mouse models have shown that colorectal adenomas can arise from both undifferentiated stem cells at the bottoms of intestinal crypts and from transit-amplifying differentiated cells, although with different disease courses, implying that the CRC phenotype and related therapeutic strategies are dependent on the cellular and molecular backgrounds of the cell of origin, as well as on cellular microenvironment [11, 12].

MicroRNAs (miRNA) are small non-coding RNA molecules (18–24 nucleotides) finely regulated by cells to induce targeted gene silencing or the post-transcriptional modulation of gene expression by base-pairing with complementary 5′, 3′ and/or other mRNA sequences. miRNAs contribute to both stem cell homeostasis in normal development and to cell fate decisions in CRC [13]. Clinical studies have also confirmed their utility as molecular diagnostic and prognostic biomarkers for a wide range of human diseases, including CRC [14–16]. Some evidences suggest that age, gender and other parameters could affect miRNA expression. Moreover, oncogenic miRNAs (oncomiR) can be selectively enclosed in and accurately released from bloodstream exosomes derived from the exocytosis of cancer cells [17] and these cancer-derived miRNA-filled exosomes have transformation and/or metastatic potential, including at distant sites [18], as well as clinical relevance [19–22]. Hence, miRNAs identification in cancer tissues and body fluids potentially have great clinical utility in early diagnosis and prognosis. However, the translation of miRNA-based knowledge to clinical practice in the treatment of CRC has not yet been achieved, and the impact of miRNAs on stem cell and colorectal CSC biology has emerged only recently [23–29]. Moreover, many reports in the scientific literature have been either *in vitro* or patient-based studies, and there are few studies aimed to identify colorectal CSC-associated miRNAs and their usefulness as tissue and/or serum biomarker in CRC patients.

In this study, putative CSC-associated miRNA profiles of three *in vitro* experimental colorectal CSC models and the tissues and sera of CRC patients were performed to identify miRNA biomarkers of CSCs and determine whether these CSC-associated miRNAs play a role as clinical biomarker in a group of adult CRC patients. To this end, the expression of a set of miRNAs selected from bibliographic sources, including miR-21, miR-221, miR-18a, miR-210, miR-31b, miR-34a, miR-10b and miR-16, was for the first time investigated with quantitative PCR (qPCR) in three *in vitro* colorectal CSC models generated after enrichment from the established CRC cell lines HCT-116, HT-29 ad T-84 by the patented protocol WO2016020572A1. The same set of miRNAs was then evaluated in a group of adult CRC patients, in whom their expression was evaluated in cancerous tissues (CT) and in ultrapurified serum (S), after normalization to the miRNA content of healthy tissues (HT, healthy colonic frustules collected in proximity of CT). The statistical relationships between the expression of miRNAs and clinical parameters, including cancer grade and presence of metastasis, as well as biochemical data, were established.

## RESULTS

### CSC enrichment in established human CRC cell lines HCT-116, HT-29 and T-84

**Figure 1:**
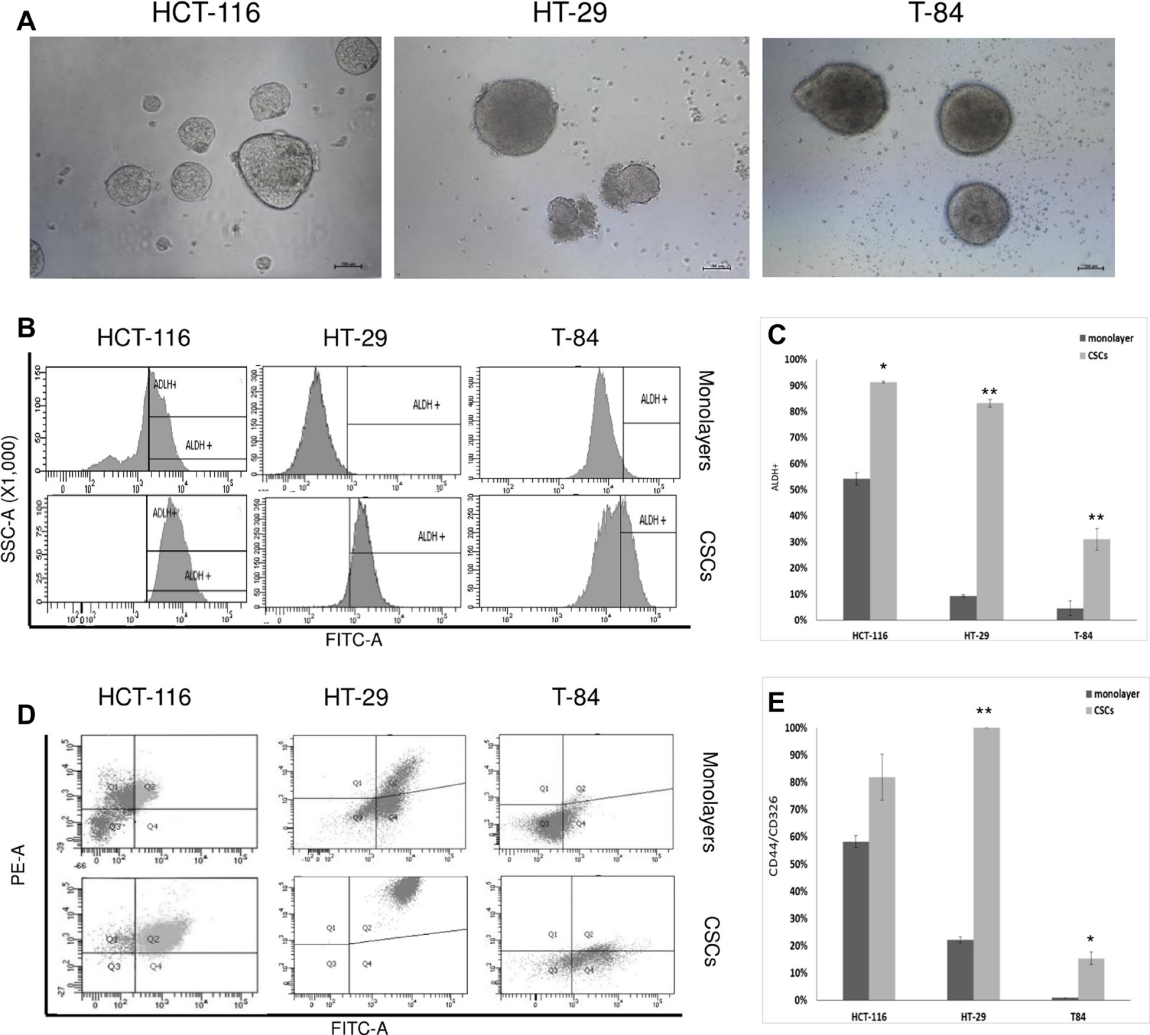
CSC enrichment of HCT-116, HT-29 ad T-84 cells and flow cytometric analyses of ALDH1 and CD44/CD326. (**A**) Secondary colonospheres enriched in CSCs after 6 days of cultures; bar = 100 μm. (**B**) Flow citometry histograms of ALDH1 activity in CSCs and monolayers; *x-axis*: FITC-A/ALDH1, *y-axis*: SSC-A/side scatter. (**C**) Statistical analysis of ALDH1 activity in CSCs ad monolayers; ^*^ *p <* 0.05, ^**^ *p <* 0.01. (**D**) Representative flow cytometry cytogram plot of CD44/CD326 ratio in CSCs ad monolayers *x-axis*: FITC-A/CD326, *y-axis*: PE-A/CD44, Q1: CD44^+^/CD326^-^, Q2 CD44^+^/CD326^+^, Q3: CD44^-^/CD326^-^, Q4 CD44^-^/CD326^+^. (**E**) Statistical analysis of CD44/CD326 in CSCs and monolayers; ^*^ *p <* 0.05, ^**^ *p <* 0.01.

Cancer stem cell enrichment was achieved in the HCT-116, HT-29 and T-84 CRC cell lines after secondary colonosphere formation. The colonospheres reached a diameter (ø) > 200 μm after 4 days of CSC enrichment and the cell pellets were collected from the secondary colonospheres on day 6 (Figure 1A). The CSC marker aldehyde dehydrogenase (ALDH1) was evaluated with flow cytometry in cells derived from colonospheres and compared to ALDH1 in the cell lines cultured in adherent condition (monolayers, Figure 1B). ALDH1 activity increased significantly from 54.2% in the HCT-116 monolayer to 91.3% in the HCT-116 colonospheres (*p <* 0.05); from 9.25% in the HT-29 monolayer to 83.2% in the HT-29 colonospheres (*p <* 0.005); and from 4.5% in the T-84 monolayer to 31% in the T-84 colonospheres (*p <* 0.005) (Figure 1C). The positivity to CD44/CD326 was also determined (Figure 1D), and increased from 58% in the HCT-116 monolayer to 85.2% in the HCT-116 colonospheres, but with no statistical significance (*p >* 0.05); from 22.2% in the HT-29 monolayer to 100% in the HT-29 colonospheres (*p <* 0.005) and from 0.9% in the T-84 monolayer to 15.4% in the T-84 colonospheres (*p <* 0.005) (Figure 1E). Isolated cells from ALDH1 and CD44/CD326 positive colonospheres are hereafter referred to as “CSC”.

### Comparison of miRNAs in colorectal CSC and monolayer-cultured cell lines, and the normal colonic cell line CCD-18Co

The set of miRNAs was evaluated in an *in vitro* model able to distinguish cancer-related miRNAs (both stem and non stem) from miRNAs of healthy cells. To this end, the Ct values of the miRNAs in the three CSCs and the corresponding monolayer cells were normalized to the miRNA contents of the CCD-18Co human colonic fibroblast cell line, which was included in the study as a healthy non-stem-cell and non-cancer-cell *in vitro* reference model.

**Figure 2:**
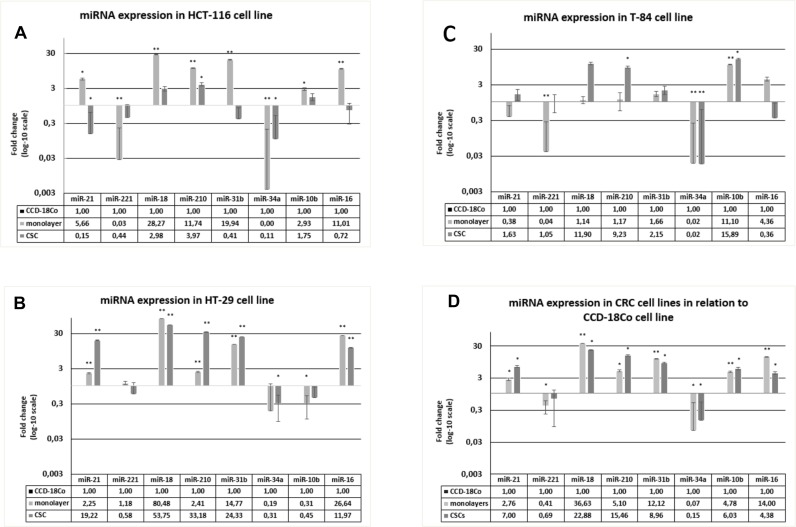
Expression of miRNAs in HCT-116, HT-29 and T-84 CSCs and monolayers, in relation to the expression in CCD-18Co cells. (**A**) miRNAs expression in HCT-116 CSC and monolayer normalized on CCD-18CO. (**B**) miRNAs expression in HT-29 CSC and monolayer normalized on CCD-18CO. (**C**) miRNAs expression in T-84 CSC and monolayer normalized on CCD-18CO. (**D**) Average of miRNAs expression in CSCs and monolayer normalized on CCD-18CO. ^*^ *p <* 0.05, ^**^ *p <* 0.01.

The fold change (FC) values for miR-18a (*p <* 0.05 for monolayer), miR-210 (*p <* 0.05 both in CSC and monolayer) and miR-10b (*p <* 0.05 for monolayer) were highest in CSC and/or monolayer of the HCT-116 model, whereas those for miR-221 (*p <* 0.05 for monolayer) and miR-34a (*p <* 0.05 both in CSC and monolayer) were highest in the CCD-18Co cells. In contrast, miR-21 was higher in the HCT-116 monolayer and lower in the CSC than in healthy cells (*p <* 0.05 for both CSC and monolayer). Similar patterns were also observed for miR-31b and miR-16, but were only statistically significant in the monolayer (Figure 2A). In the HT-29 model, miR-21, miR-18a, miR-210, miR-31b and miR-16 were higher in the CSC and monolayer than in the CCD-18Co cells (*p <* 0.05), whereas miR-34a and miR-10b were higher in the CCD-18Co cells than in the CSC or monolayer (*p <* 0.05). miR-221 was slightly downregulated in the HT-29 CSC and upregulated in the monolayer relative to its level in healthy cells, but the differences were not statistically significant (Figure 2B). In the T-84 model, miR-210 (*p <* 0.05 in CSC) and miR-10b (*p <* 0.05 in CSC and monolayer) were higher in the CSC and/or monolayer than in healthy cells, whereas miR-34a was higher in the CCD-18Co cells than in the T-84 cells (*p <* 0.05 for both CSC and monolayer). miR-221 was significantly lower in T-84 monolayer than in healthy cells, whereas miR-16 was higher in the T-84 monolayer but lower in the CSC than in healthy cells, although the differences were not statistically significant (Figure 2C). From a global perspective, we also merged the results for the three *in vitro* models of colorectal CSC to determine the average FC values for the miRNAs in the CSCs and monolayers, and analysed them statistically. miR-21 (*p =* 0.03 for monolayers, *p =* 0.02 for CSCs), miR-18a (*p =* 0.001 for monolayers, *p =* 0.01 for CSCs), miR-210 (*p =* 0.01 for monolayers, *p =* 0.01 for CSCs), miR-31b (*p =* 0.01 for monolayers, *p =* 0.02 for CSCs), miR-10b (*p =* 0.01 for monolayers, *p =* 0.03 for CSCs) and miR-16 (*p =* 0.007 for monolayers, *p =* 0.038 for CSCs) were globally associated with the cancer phenotype, whereas miR-221 (*p =* 0.019 for monolayers, *p >* 0.05 for CSCs) and miR-34a (*p =* 0.01 for monolayers, *p =* 0.02 for CSCs) were usually associated with the healthy fibroblast phenotype (Figure 2D).

### miRNA expression in colorectal CSC and monolayer cell lines

**Figure 3:**
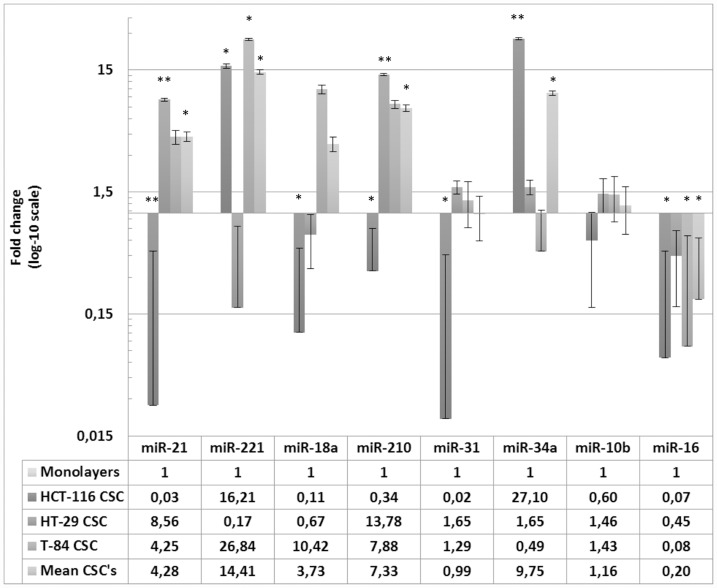
Expression of miRNAs in HCT-116, HT-29, T-84 CSCs in relation to the expression in respective monolayers. Data are shown for CSC separately and as average of miRNAs expression in CSCs normalized to monolayers ^*^ *p <* 0.05, ^**^ *p <* 0.01.

To highlight a CSC-associated miRNA pattern from the one of cells routinely cultured in adherent conditions, the differences in the expression of the miRNAs between the three CSCs and corresponding monolayer cells were also determined, and showed significant changes (Figure 3). In HCT-116 CSC, the most strongly expressed miRNAs were miR-34a (FC = 27.09; *p =* 0.0071) and miR-221 (FC = 16.20; *p =* 0.0231), whereas the other miRNAs were all downregulated (miR-21, miR-18a, miR-31, miR-16; all *p <* 0.05). In the HT-29 CSC, the most strongly expressed miRNAs were miR-210 (FC = 13.78; *p =* 0.0056) and miR-21 (FC = 8.55; *p =* 0.0047), together with miR-31, miR-34a and miR-10b, although the changes in these cases were not statistically significant (*p >* 0.05). Similarly, miR-221, miR-18a and miR-16 were downregulated, although not statistically significantly, so no robust miRNA downregulation occurred in HT-29 CSC relative to their expression in the monolayer HT-29 cells. In the third *in vitro* model, T-84 CSC showed the highest expression of miR-221 (FC = 26.84; *p =* 0.0138) and miR-210 (FC = 7.87; *p =* 0.0374). Although miR-18a (FC = 10.42; *p =* 0.0575) and miR-21 (FC = 4.24; *p =* 0.0618) also had high FC values, the changes were not statistically significant because the standard errors were high. The uniquely downregulated miRNA in T-84 CSCs was miR-16 (FC = 0.08; *p =* 0.0245), whereas the differences in miR-31, miR-34a and miR-10b expression between the T-84 CSC and monolayer cells were not statistical significant.

When all three CSC models were considered together (Figure 3), the CSCs-related miRNAs most strongly upregulated were miR-21 (FC = 4.27; *p =* 0.0355), miR-221 (FC = 14.40; *p =* 0.0233), miR-210 (FC = 7.33; *p =* 0.0216) and miR-34a (FC = 9.74; *p =* 0.0146), whereas miR-18a, miR-31 and miR-10b showed similar expression to that in the three monolayers. Interestingly, miR-16 (FC = 0.19; *p =* 0.0319) was the only significantly downregulated miRNA in all three CSC models, considered alone or together (no expression), whereas it showed the basal level of expression in the monolayer cells.

### miRNA expression in cancer tissues and ultrapurified sera from CRC patients

**Figure 4:**
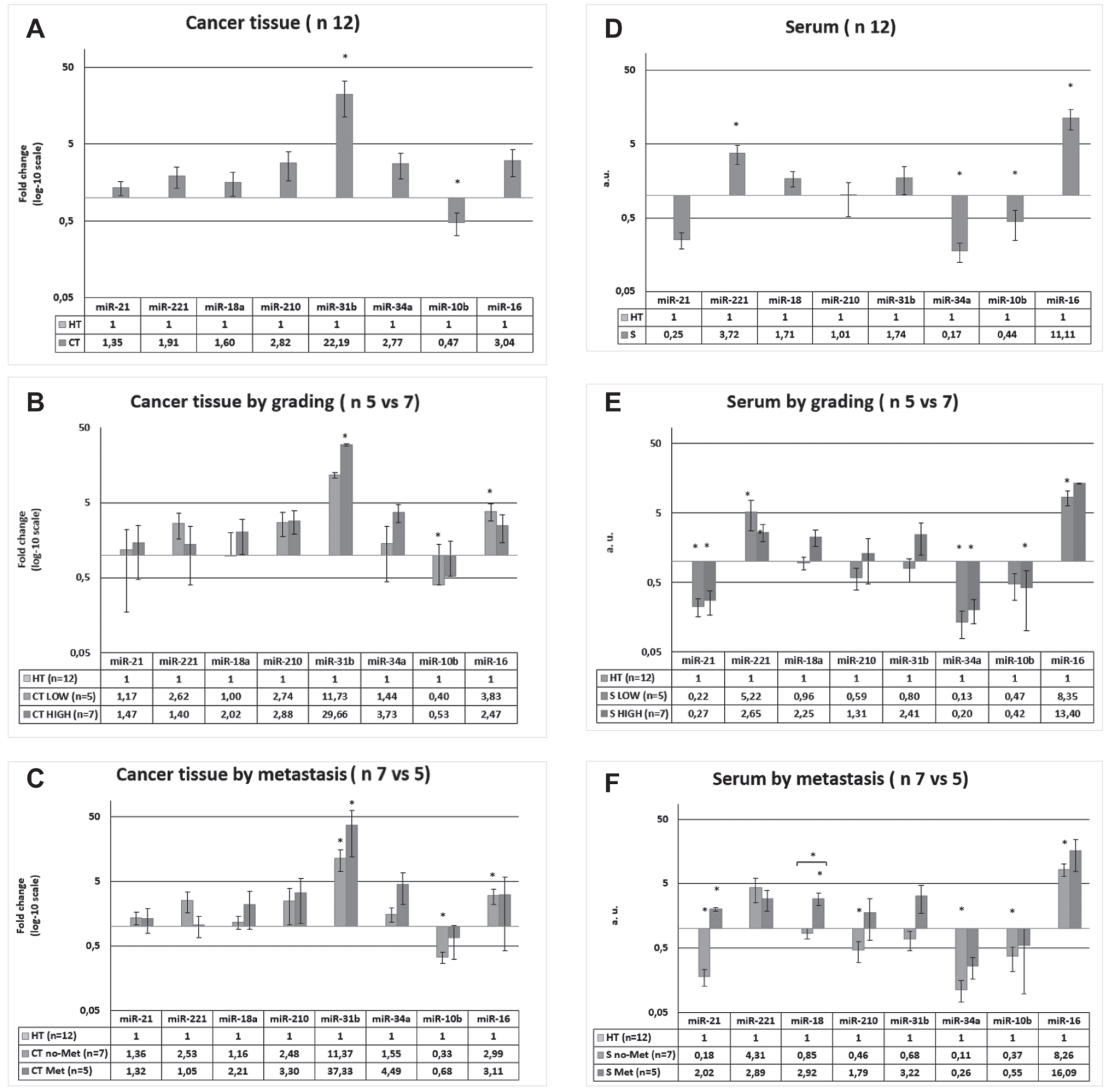
Expression of miRNAs in cancer tissue (CT) and serum (S) of CRC patients. The data of CT ad S were both normalized to healthy tissue in each patients. (**A**) miRNAs level in CT vs HT of 12 patients. (**B**) miRNAs level in CT vs HT in patients divided in two groups in function of patients’ grade. (**C**) miRNAs level in CT vs HT in patients divided in two groups in function of metastasis. (**D**) miRNAs level in S vs HT of 12 patients. (**E**) miRNAs level in S vs HT in patients divided in two groups in function of patients’ grade. (**F**) miRNAs level in S vs HT in patients divided in two groups in function of metastasis. ^*^ *p <* 0.05, ^**^ *p <* 0.01.

All the miRNAs investigated, with the exception of miR-10b, were upregulated in the cancer tissues (CT) of all CRC patients (*n =* 12) relative to their expression in healthy tissues (HT), but the difference was only significant for miR-31b, which was highly upregulated (FC = 22.19; *p =* 0.0019), and for miR-10b, which was significantly downregulated (FC = 0.47; *p =* 0.0088) (Figure 4A). After the patients were grouped into two categories according to their tumour grade (LOW: stages 0–IIIA, *n =* 5; or HIGH: stages IIIB–IVA, *n =* 7), the same CT–HT analysis showed that miR-31b was upregulated and miR-10b downregulated significantly more often in HIGH patients and in LOW patients, respectively, and that miR-16 acquired significance in LOW patients (FC = 3.83; *p =* 0.0025), and not in the total group of patients or in the HIGH group (Figure 4B). When the patients were grouped by metastasis (NO, *n =* 7; YES, *n =* 5), a similar pattern was observed, except that miR-31b was also significantly upregulated in no-Met patients (*p <* 0.05; Figure 4C).

The miRNA analysis in ultrapurified serum (S), with a methodology similar to that used to determine the serum exosome patterns, produced results in arbitrary units (a. u.) for each miRNA. It showed that miR-221 (a. u. = 3.72; *p =* 0.0046) and miR-16 (a. u. = 11.11; *p =* 0.003) were upregulated and that miR-34a (a. u. = 0.17; *p =* 0.0015) and miR-10b (a. u. = 0.44; *p =* 0.0277) were downregulated in S from the CRC patients relative to their expression in the corresponding HT (Figure 4D). Both miR-18a and miR-31b were upregulated and miR-21 was downregulated in S, but the differences were not statistically significant. After the patients were divided in two groups according to tumour grade (LOW, *n =* 5; HIGH, *n =* 7), the S–HT analysis showed significant upregulation of miR-221 and miR-21 and significant downregulation of miR-34a in both the LOW and HIGH groups of patients relative to the controls, and miR-16 upregulation (a. u. = 8.35; *p =* 0.0002) and miR-10b downregulation (a. u. = 0.42; *p =* 0.0077) specifically in the LOW patient group (Figure 4E). When the patients were grouped by metastasis (no-Met = 7; Met = 5), the following S–HT differences were observed. In Met patients, only miR-21 (a. u. = 2.02; *p =* 0.0156) and miR-18a (a. u. = 2.92; *p =* 0.0175) were significantly higher in S than in HT, whereas in no-Met patients, miR-21 (a. u. = 0.18; *p =* 0.0001), miR-210 (a. u. = 0.46; *p =* 0.0127), miR-34a (a. u. = 0.11; *p =* 0.0001) and miR-10b (a. u. = 0.37; *p =* 0.0072) were lower in S than in HT, and both miR-221 (a. u. = 4.31; *p =* 0.0023) and miR-16 (a. u. = 8.26; *p =* 0.0001) were higher in S than in HT (Figure 4F). Moreover, the miR-18 levels in S were higher in Met patients than in the no-Met patients (a. u = 3.43; *p =* 0.0059; Figure 4F).

A CT–S comparison was also performed, in which miR-31b expression was significantly higher in CT than in S in the 12 CRC patients, and was also significantly higher in CT than in S in the LOW (*p =* 0.0265), HIGH (*p =* 0.0056), no-Met (*p =* 0.0021) and Met (*p =* 0.0339) patients taken alone. According to TNM, miR-21 (*p =* 0.0204), miR-31b (*p =* 0.0265) and miR-34a (*p =* 0.003) where higher in CT than in S in the LOW patients, whereas no other differences were observed in the HIGH patients. In relation to metastasis, only miR-31b (*p =* 0.0399) was higher in CT than in S in Met patients, whereas miR-21 (*p =* 0.0007), miR-210 (*p =* 0.0334), miR-31b (*p =* 0.0021) and miR-34a (*p =* 0.0001) where higher in CT than in S in the no-Met patients. This differed from the pattern for miR-16, whose expression was higher in S than in CT in no-Met patients (*p =* 0.0275).

### Spearman’s correlation analysis of miRNAs and clinical parameters

Spearman’s correlation matrices between the miRNA levels in CT or S and clinical parameters identified a set of significant positive and negative correlations. In CT, miR-221 correlated positively with sex and negatively with aspartate aminotransferase (AST); in S, miR-221 correlated positively with age and negatively with both alanine aminotransferase (ALT) and (AST). In CT, miR-18a correlated positively with low-density lipoprotein (LDL) and negatively with age; in S, miR-18a correlated positively with metastasis and triglycerides. In CT, miR-34a correlated positively with alkaline phosphatase (ALP; Table 1). The correlations between the different variables and miR-21, miR-210, miR-31b, miR-10b and miR-16 (CT or serum) and miR-34a (S) were not significant (not shown).

**Table 1:** Spearman‘s rank correlation coefficients between miRNA levels in patient’s cancer tissues (CT) or serum (S) and demographic/clinical variables

	miR-221		miR-18a		miR-34a
	CT	S	CT	S	CT
Age	−0,0866	0,6879^*^	−0,6651^**^	0,4009	0,3554
Sex	0,7769^*^	0,5379	0,0000	−0,3586	−0,3586
Met	−0,4781	−0,3586	0,1793	0,6574^*^	0,0598
GGT1	−0,5818	−0,4636	0,2091	0,4818	0,3091
ALT/GPT GPT?	−0,5138	−0,6055^**^	0,0459	−0,1560	0,0183
AST	−0,7002^**^	−0,6362^**^	0,1098	0,3341	0,1327
CPK	−0,0091	−0,3455	-0,0364	−0,0818	−0,1455
ALP	−0,4000	0,0455	-0,2364	0,4364	0,6091^*^
CHOL	0,5000	−0,0636	0,5545	−0,2545	−0,4455
HDL	0,1727	0,2182	0,0182	−0,5818	−0,4000
LDL	0,5057	−0,3462	0,6743^*^	−0,1321	−0,4191
TG	−0,2636	−0,0091	0,2182	0,6455^*^	−0,0091
GLY	−0,3319	−0,0935	−0,0795	0,1122	−0,4207

Abbreviations: Age, sex, metastasis (MET), gamma-glutamyl transferase (GGT1), alanine transaminase (ALT or GPT?), aspartate transaminase (AST), creatine phosphokinase (CPK), alkaline phosphatase (ALP), cholesterol (CHOL), high density lipoprotein (HDL), low density lipoprotein (LDL), trygliceride (TG), glycemia (GLY). The correlation matrixes of miRNA with no correlation with any of the variables considered are not shown. ^*^ significant positive correlation (*p <* 0,05); ^**^ significant negative correlation (ρ < 0,05).

## DISCUSSION

We examined putative colorectal CSC-associated miRNAs both *in vitro* and in CRC patients, in three different CSC models obtained from human established CRC cell lines and CT, HT and S of adult CRC patients, respectively. We then correlated the miRNA levels with clinical data.

Evaluating *in vitro* the miRNAs in a) HCT-116, HT-29 and T-84 colorectal CSC models; b) HCT-116, HT-29 and T-84 monolayer-cultured cell lines; and c) the normal colonic cell line CCD-18Co allowed us to distinguish the miRNAs specific to the cancer cell phenotype in stem and non-stem cancer cells and to normal colon cells. In some cases, the three CSC models showed different miRNA profiles, and these profiles also differed from those in the corresponding monolayers-cultured cells. These miRNAs were highly deregulated in the CT and S of CRC patients, suggesting new options for the development of miRNA-directed clinical tools.

miR-21 is known to target *PTEN* oncosuppressor gene transcripts, the downstream effectors of which promote EMT, cell migration and the invasion of CRC organoids by inhibiting TIAM1 [30]. Here, we selected miR-21, together with miR-221, as the positive control for the miRNA assays because its function is also linked to the self-renewal control in CSC and the reduction of AKT phosphorylation in CRC [31]. According to our data, miR-21 was upregulated in CSCs and in the samples of CRC patients, with the only exception being HCT-116 CSC. This last result looks in contrast with other studies, in which miR-21 was upregulated in CSC derived from HCT-116 cells, but using different CSC-enrichment methods and markers [13, 32–34]. We also showed that miR-221 was upregulated in HCT-116 and T-84 CSCs, as well as in CT and S of CRC patients. This result is consistent with other studies that have reported the role of miR-221 in cancer cell proliferation by inhibiting CDKI, which promotes G0/G1-to-S cell-cycle phase advancement [35]. In contrast, miR-221 was downregulated in HT-29 CSC, consistent with another study that showed miR-221 downregulation in CD133^+^ HT-29 CSC relative to its expression in differentiated CD133^-^ HT-29 cells [36]. Therefore, we suggest that HT-29 cells could adopt other CDKI regulatory mechanisms in cell-cycle progression. Surprisingly, CCD-18Co cells showed higher miR-221 expression than HCT-116 or T-84 cells, suggesting that miR-221 also plays a role in the replication of normal colon cells. miR-221 was also upregulated in S from CRC patients, and more significantly in the LOW and no-Met patients, suggesting that miR-221 acts early in CRC. It was positively correlated with age, suggesting a possible CRC initiation biomarker role, especially in younger patients. Therefore, miR-221 may offer a tool for the blood-based early screening of CRC.

miR-18a is part of the 17-92 cluster, the last known for its oncogenic role in CRC [37], and its upregulation has been described in several tumours [38, 39], including CRC [37]. In contrast, other authors have demonstrated that miR18a plays a balancing role in the 17–92 cluster, acting as an anti-oncomiR (miRNA with ocosuppressor activity) that inhibits CDC42 [40]. It may also target the transcripts of the *KRAS* oncogene, which is involved in the initiation and progression of CRC [41]. Here, we showed that miR-18a was upregulated in all cancer models, both *in vitro* and in patients. However, it was downregulated in HCT-116 and HT-29 CSCs relative to its expression in the corresponding monolayers, consistent with the work of Tsang *et al*. (2009), who reported that miR-18a suppression increases HT-29 cell proliferation and anchorage-independent growth [41]. Therefore, we suggest that the loss of miR-18a function could induces KRAS activation, which may play a key role in the stemness of HCT-116 and HT-29 cells. miR-18a was also dramatically upregulated in patients, especially significantly in S of Met patients. The last insightfully result is strongly corroborated by the positive correlation with metastases in Spearman’s correlation analysis, that showed also a negative correlation of miR-18a with age, suggesting that high level of miR-18a in blood of adult patients, rather than in younger patients, could be indicative of the presence of CRC metastasis. Thereby supporting a potential role for miR-18a as a potent serum-based cancer biomarker [42], especially valid for adult CRC metastatic patients. However, appropriate mechanistic studies are required to clarify the real function of this miR in the stemness properties of cells and in cancer progression and metastasis.

miR-210 plays an important role in hypoxia [43] and is known to induce apoptosis in colorectal cells, including HCT-116 cells [44], and it is also involved in cell migration and invasion, promoting CRC metastasis [45]. Here, we showed that miR-210 is upregulated in CSCs relative to its expression in the corresponding monolayers (with the exception of HCT-116) and in CT relative to its expression in HT. This is consistent with others studies that reported miR-210 upregulation in adenocarcinomas relative to its expression to the controls, and correlation with CRC TNM and clinical stage [46]. The upregulation of miR-210 in T-84 CSC looks in contrast with the results of Tsuchiya *et al*. (2009), who reported that miR-338, miR-451, miR-210 and miR-33a are upregulated in T-84 cell line committed to cell differentiation by the translocation of basolateral membrane β1-integrin. However, they also demonstrated mechanistically that miR-210 upregulation plays no role in the differentiation of T-84 cells [47]. In accordance with the authors, miR-210 upregulation in T84 CSC observed in this study coexist with a state of cell stemness. We also observed the downregulation of miR-210 in HCT-116 CSC, in line with the findings of Tagscherer *et al*. [44], which could represent an apoptosis-avoiding molecular mechanism, consistent with the apoptosis-inducing role of miR-210 in these cells.

miR-31 has oncosuppressor activity in a number of cancer cells, even when there are defects in the TP53 pathway. However, in CRC it is linked to disease progression, particularly in the non-conventional pathway of serrated colorectal lesions, which requires the mutation or deregulation of BRAF, but not of APC [48]. Here, we showed that miR-31 is upregulated in cancer cells relative to its expression in healthy cells, and in CSCs relative to its expression in monolayers, with the only exception being in HCT-116 CSC. These trends are consistent with studies that have demonstrated roles for miR-31 loss of function in chemoresistance, cell migration and invasion, but not in the proliferation of HCT-116 cells [49]. This differs from its role in BRAF- and TP53-mutated HT-29 cells, in which its function is closely associated with cell proliferation [50]. Slight miR-31 downregulation in CD133^+^ HT-29 cells with stem-like properties was reported by Zhang *et al.* [36], in contrast to our data for HT-29 CSC. However, the usefulness of CD133 as a unique marker of colorectal CSC is debated for a number of cancer cell types [51, 52]. In our patients, miR-31 was upregulated in both CT and S, with the highest expression in the HIGH and Met patients, suggesting that miR-31 is a candidate CRC-specific diagnostic and prognostic marker, as well as a druggable therapeutic target in clinical practice.

Dramatic reductions in miR-34a expression were observed in all cancer models, corroborating the bond between miR-34a and both NOTCH1 [53, 54] and WNT deregulation [55], two major pathways in CRC and colorectal CSC of various origin. However, we also showed unexpectedly lower miR-34a expression in the HCT-116 and HT-29 cell lines relative to its expression in HCT-116 and HT-29 CSCs, respectively, suggesting possible WNT pathway activity also in the monolayer cell cultures. However, in the T-84 CSC the expression of miR-34a was high, as expected. In our patients, no differences in miR-34a expression were detected between CT and HT, but there was only an increasing trend in CT, while miR-34a levels were significantly lower in S than in HT.

The expression and function of miR-10b have received little attention, but some authors have reported that the loss of miR-10 induces WNT signalling [56] and that stem-like cells in head-and-neck cancer recruit miR-10b to promote chemoresistance, cell migration and invasion [57]. In our *in vitro* CSC models, miR-10b was slightly deregulated in relation to its expression in monolayer cells, with its highest expression in T-84 CSC. However, in this study miR-10b was also the most significantly downregulated miRNA in CT and S in the LOW group and in both Met and no-Met patients, suggesting that its early downregulation is associated with CRC initiation and progression.

The downregulation of miR-16 occurs in a number of solid tumours, including CRC, and is associated with the inhibition of cell proliferation by modulating the TP53/survivin pathway [58]. Triggering its expression in CRC can lead to cancer inhibition *via* the intrinsic apoptosis pathway [59]. Consistent with this scenario, we clearly demonstrated that miR-16 expression was dramatically downregulated in all the CSC models of this study. In contrast, miR-16 was upregulated in CT and S, particularly in the LOW grade and no-Met patients, suggesting that high level of miR-16 in cancer tissues is an early diagnostic marker of CRC. This is consistent with several studies that have reported high levels of miR-16 in CRC tissues [60] and/or its association with overall survival [61, 62], but in contrast to other studies that have reported its downregulation in CRC [63]. Because this miRNA was downregulated in all CSC types, its reduced expression in patients with CRC could be a marker of poor prognosis.

In conclusion, a set of CSC-associated miRNAs were identified using different *in vitro* CRC models, and significant differences in the expression of these miRNAs and clinical correlation were detected in tissues and serum of adult CRC patients stratified according to cancer grade and metastatic status. Based on these results, which emerged from a little but targeted number of patients according to TNM stage and metastasis, the major limit of this study, we suggest that CRC biopsy and serum samples contain both stem and non-stem miRNAs, some of which could play roles as diagnostic and prognostic factors in the clinical management of CRC and as potential therapeutic targets. Our results offer new data supporting the idea that miRNAs have utility in clinical practice and pave the way for future studies on a greater number of miRNA and patients with the aid of gold standard methodologies such as microarray and next generation sequencing to go deeply towards the identification of new miRNA-based tools for CRC diagnostic procedures, even blood-based procedures.

We have also noted several similarities in the miRNA expression patterns in CSCs and healthy colonic fibroblast cells. This last unexpected result suggests that fibroblasts, mesenchymal cells and CSCs share some structural and molecular traits [27, 64, 65].

## MATERIALS AND METHODS

### Cell line cultures

The human colon fibroblast cell line CCD-18Co and the human CRC cell lines HCT-116 (with mutations in *CTNNB1*, *KRAS*, *PIK3CA*, *TGFR2*, *CDKN2A* and *BRCA2*), HT-29 (with mutations in *APC*, *BRAF*, *PIK3CA*, *SMAD4* and *TP53*) and T-84 (with mutations in *KRAS* and *TP53*) were purchased from the American Type Culture Collection (ATCC, 10801 University Boulevard Manassas, VA 20110 USA) and cultured under standard conditions in Dulbecco’s modified Eagle’s medium (DMEM) containing 10% of foetal bovine serum (FBS) and 1% penicillin/streptomycin (Pen-Str P-0781, Sigma) at 37° C under 5% CO_2_.

### CSC enrichment

Colorectal CSCs were obtained from HCT-116, T-84 and HT-29 cells with the patented protocol WO2016020572A1 [66], which relies on the generation of colonospheres in Corning^®^ Costar^®^ Ultra-Low Attachment Six-Well Plates in DMEM/F-12 nutrient mixture without serum, supplemented with 1× B-27 (B-27™ Supplement [50×], Minus Vitamin A; Invitrogen), 4 ng/mL heparin (heparin sodium cell culture tested, Sigma), 10 μg/mL insulin (Insulin-Transferrin-Selenium [ITS-G] [100×], Invitrogen), 1 μg/mL hydrocortisone (Sigma), 10 ng/mL epidermal growth factor (Sigma), 10 ng/mL fibroblast growth factor (Sigma), 10 ng/mL interleukin 6 (Miltenyi) and 10 ng/mL hepatocellular growth factor (Miltenyi). After 72 h, the colonospheres were disaggregated by incubation with trypsin (T-4049; Sigma) at 37° C. The trypsin was inactivated by the addition of DMEM containing serum. The colonospheres were washed with phosphate-buffered saline (PBS) to remove traces of FBS, and the single cells were resuspended in new low-attachment plates with serum-free medium to generate secondary colonospheres.

### Flow-cytometric analyses

ALDEFLUOR™ assays (Stem Cell Technologies) were used to detect ALDH1 activity, according to the manufacturer’s instructions. Diethylaminobenzaldehyde was used as an ALDH1 inhibitor to set the ALDH1 gates. The cell-surface levels of CD44 and CD326 were determined with anti-human CD44–phycoerythrin and CD326–fluorescein isothiocyanate antibodies (Miltenyi Biotec). All samples were analysed with a FACSCanto™ II flow cytometer (BD Biosciences) using the FACSDiva™ software.

### miRNA set selection

The set of candidate miRNAs was chosen after a comprehensive literature search of miRNAs with roles as oncomiRs (miR21, miR-221, miR-210 and miR-18) or anti-oncomiRs (miR-34, miR-10b and miR-16) in CRC. miR-31 was included for its pleiotropic role in cancer, and miR-24 was used for data normalization.

### RNA extraction from cells

Cells were disaggregated with trypsin, pelleted at 1500 × g for 5 min, and washed twice in cold PBS. Then 1 ml of TRI Reagent^®^ (Sigma-Aldrich) was added to the pellets and the cells were transferred to 1 ml Eppendorf microtubes and allowed to stand at room temperature (RT) for 15 min. After chloroform (200 μl) was added, the cells were vortexed for 15 s and left to stand at RT for 10 min. The cells were centrifuged at 12,000 × g for 10 min at 4° C, and the upper aqueous phase of the solution was transferred into a new Eppendorf microtube. Isopropanol (500 μl) was added to the aqueous phase, which was then vortexed, incubated at RT for 10 min, and centrifuged at 12,000 × g for 10 min at 4°C. The isopropanol was removed and replaced with 1 ml of ethanol (EtOH, 75% in Milli-Q™ water) before centrifugation at 17,000 × g for 5 min at 4° C. After the EtOH was removed, the sample was left at RT for 1 h and then resuspended in 50 μl of Milli-Q™ water. The RNA concentrations and quality were evaluated routinely with a NanoDrop spectrophotometer (Thermo Fisher Scientific).

### RNA extraction from patients’ biopsy

Informed consent for the use of human material for research was obtained from the patients before sampling. The Institutional Medical Ethics Committee of the University of Sassari (V. le S. Pietro 43/C, 07100 Sassari, Italy) approved the study. To establish the cancer staging by histological methods, the 0-IV TNM classification is highly recommended according the AIOM guidelines 2018, where “T” denotes the degree of invasion of the intestinal wall, “N” the degree of lymphatic node involvement, and “M” the presence or absence of metastasis. The severity of CRC was classified according to TNM and three kind of samples were collected from 12 patients: CRC tissue (CT), healthy colorectal tissue (HT) and peripheral blood for serum ultrapurification (S).

Before surgery or any treatment, 5 ml of blood was collected from the patients and transferred to Serum-Gel Clotting Activator S-Monovette™ (9 ml tube, Sarstedt), incubated at RT for 60 min, and ultracentrifuged to obtain ultrapurified serum aliquots. During the surgical removal of the tumour mass, samples of colorectal cancer and healthy biopsy tissue were obtained from the CRC patients (U. O. General Surgery I, Surgical Pathology AOU Sassari, University of Sassari), and immediately transferred in RNA*later*-filled 15 ml tubes (Sigma). After 24 h at 4° C, the RNA*later* was removed and the dried samples were stored at −80° C until processing. The cancerous and healthy tissues (30 mg) were cut into thin pieces with a scalpel in Petri’s dishes and transferred into 2 ml vials containing 700 μl of QIAzol Lysis Reagent (Qiagen) and one stainless steel bead (ø = 7 mm). The tissues were homogenized with a TissueLyser LT (Qiagen) at 40 oscillations/min for 2 min. The RNA was extracted by miRNeasy^®^ Mini Kit (Qiagen), according to the manufacturer’s instructions. The RNA concentrations and quality were determined with a NanoDrop spectrophotometer. The blood samples were centrifuged at 1900 × g for 10 min at 4° C. The serum in the upper phase was transferred to new tubes and centrifuged at 16,000 × g for 10 min at 4° C. The supernatant was transferred to a new 1.5 ml tubes and stored at −80° C until processing. After the samples were thawed at RT for 5 min, five volumes of QIAzol Lysis Reagent were added to each sample and incubated at RT for 5 min, before the miRNAs were extracted by miRNeasy^®^ Serum/Plasma Kit (Qiagen), according to the manufacturer’s instructions.

### Real-time reverse transcription (RT)–PCR assay

The RNA samples were diluted in nuclease-free water and resuspended to a final concentration of 5 ng/μl. cDNA was synthesised with the miRCURY™ LNA™ RT Kit (Qiagen), according to the manufacturer’s instructions. The reactions were spiked with exogenous UniSp6 RNA (RNA Spike-In Kit, Qiagen). The following RT protocol was applied: 60 min at 42° C, 5 min at 95° C; immediate cooling to 4° C; storage at −20° C until processing.

The Real-Time LNA™ PCR primer set (Qiagen) was used as follows: hsa-miR-221-3p for mir-221, hsa-miR-34a-5p for miR-34a, hsa-miR-10b-5p for miR-10b, hsa-miR-18a-5p for mir-18a, hsa-miR-210-3p for miR-210, hsa-miR-31-5p for mir-31, hsa-miR-16-3p for mir-16, and hsa-miR-21-5p for mir-21. For data normalization and assessment of cDNA quality, we used hsa-miR-24-3p for mir-24 [67] and the UniSp6 primer set, respectively. In the Real-Time PCR assays, the cDNA was diluted 1:40 in nuclease-free water and 4 μl of diluted cDNA was mixed with 5 μl of PCR master mix and 1 μl of each primer pair. The thermal cycling conditions included a melting curve analysis and were applied as follows: one cycle at 95° C for 10 min, followed by 45 cycles at 95° C for 10 s and 60° C for 1 min, with a ramp rate of 1.6° C/s.

### Statistical analysis

In the *in vitro* experiments, we first compared the CRC cells (both CSCs and monolayers) with the normal CCD18Co cell line. We then directly compared the CSCs with the corresponding cell lines of origin cultured as monolayers. To determine the most frequent CSC-associated miRNAs, their expression in the three CSC models was analysed as the geometric means of the fold change (FC) values. Analysis of the PCR dataset was performed with the software supplied with the 7500 Real Time PCR System (Applied Biosystems). The real-time RT–PCR assays were run in triplicate and the mean cycle threshold (Ct) value for each miRNA under each set of conditions was used to determine FC with the 2^-ΔΔCt^ method, using the Ct values for the hsa-miR-24 reference miRNA in data normalization. The FC values were compared with Student’s independent *t*-test. A two-sided *p* value of < 0.05 was considered statistically significant.

In each patient, we compared the miRNA levels in the serum (S) and cancer tissue (CT) with those in the healthy tissue (HT) from the same patient. The average values for replicate spots of each miRNA were background subtracted and normalized. The patients were also stratified according to cancer grade and the presence/absence of metastasis, and appropriate statistical analyses were performed and data analysed with Microsoft Excel 10. The FCs were graphed as histograms on a logarithmic-scale Y axis (base 10). All statistical analyses were performed with Stata statistical software: release 15 (StataCorp LLC, College Station, TX, USA). Descriptive statistics were primarily used in the analysis of the data. The normality of the distributions of the miRNAs was tested with the Shapiro–Wilk test, and for normally distributed data, Student’s two-sided *t*-test was used where stated. The non-parametric Mann–Whitney *U* test followed by the two-sided Dunn’s pairwise *post hoc* test based on rank sums, with the Benjamini–Hochberg adjustment of *p* values (p), was used to compare miRNA expression. Spearman’s rho was used to assess the correlations between the miRNA expression levels and the clinicopathological parameters of the patients, including metastasis, tumour grading, GGT, ALT, AST, CPK, cholesterol, HDL, LDL, triglycerides, glycaemic status, age and sex.

## Abbreviations

ALDH1: aldehyde dehydrogenase 1
ALT: alanine aminotransferase
APC: adenomatous polyposis coli protein
AST: aspartate aminotransferase
BRAF: B-RAF proto-oncogene, serine/threonine kinase
BRCA2: breast cancer type 2 susceptibility protein
CDKN2A: cyclin dependent kinase inhibitor 2A
CPK: creatine phosphokinase
CRC: colorectal cancer
CSC: cancer stem cell
CT: cancer tissue
Ct: cycle threshold
CTNNB1: beta catenin 1
EMT: epithelial-to-mesenchymal transition
GGT: gamma-glutamyl transferase
HDL: high-density lipoprotein
HIGH: high TNM grading patients
HT: healthy tissue
KRAS: KRAS proto-oncogene, GTPase
LDL: low-density lipoprotein
LOW: low TNM grading patients
Met: metastatic patients
no-Met: non metastatic patients
PIK3CA: phosphatidylinositol 3-kinase
S: ultrapurified serum
TGFR2: transforming growth factor receptor 2
TP53: P53 tumor suppressor
